# Asymptomatic Nasal Carriage of MRSA Among Romanian Medical Students: Prevalence and Sampling Technique Comparison

**DOI:** 10.3390/microorganisms14061265

**Published:** 2026-06-04

**Authors:** Mihai Octavian Dan, Victoria Aramă, Alexandru Rafila, Daniela Tălăpan

**Affiliations:** 1 Faculty of Medicine, ‘Carol Davila’ University of Medicine and Pharmacy, 050474 Bucharest, Romania; mihai-octavian.dan0720@stud.umfcd.ro (M.O.D.);; 2 ‘Prof. Dr. Matei Balș’ National Institute of Infectious Diseases, 021021 Bucharest, Romania

**Keywords:** MRSA, asymptomatic carriage, medical students

## Abstract

Introduction: Nasal colonization plays a pivotal role in Methicillin-resistant *Staphylococcus aureus* (MRSA) carriage and transmission, especially in healthcare settings. Asymptomatic carriers amongst healthcare workers (HCWs) may serve as an important source for inner-hospital transmission, besides personal increased risks of endogenous infections. Medical students are an often-overlooked part of medical staff which, while not typically included in statistics concerning HCWs, are associated with increased patient contact and exposure to healthcare-associated pathogens. This study aimed to assess MRSA carriage rate amongst clinical-year medical students in the largest Romanian medical university, in addition to identifying potential risk factors. Nonetheless, a methodological aim was incorporated, in order to evaluate the effect of nasal swab pre-moistening with sterile saline on MRSA retrieval rate. Materials and Methods: A cross-sectional study was conducted among clinical-year students from the ‘Carol Davila’ University of Medicine and Pharmacy in Bucharest, Romania. Participants completed a survey regarding potential risk factors and underwent nasal swab sampling, being randomly assigned to the two swab collection methods—dry swab or pre-moistened swab with sterile saline, randomization ensuring comparable baseline characteristics. Samples were inoculated on chromogenic MRSA agar media and incubated for 24–48 h at 35–37 °C. Isolates exhibiting characteristic growth further underwent coagulase testing, bacterial identification and methicillin resistance confirmation. Results: The study comprised 156 medical students, with an overall prevalence of asymptomatic MRSA nasal carriage of 5.76% (n = 9, 95% CI: 3.05–10.58%). No statistically significant associations were identified between MRSA carriage and hospital exposure. The prevalence of MRSA positive cultures was 5.00% (n = 4/80) among the conventional dry swab sampling subgroup, while the subgroup undergoing pre-moistened swab collection presented a 6.57% prevalence (n = 5/76), revealing no statistical significance (*p* = 0.74). Conclusions: Asymptomatic MRSA carriage among medical students in this cohort suggests the potential role of this population in intra-hospital transmission. In addition, pre-moistening the nasal swab for collection of the sample showed no statistically significant impact on MRSA recovery rates, correlating with existing literature on the topic. These findings further emphasize the need for strict adherence to infection prevention and control measures in hospitals.

## 1. Introduction

Methicillin-resistant *Staphylococcus aureus* (MRSA) stands as a major human pathogen, especially occurring in healthcare-associated infections, leading to lengthy hospital stays and increased burden of disease [1]. From an epidemiological perspective, nasal colonization remains the primary site for asymptomatic carriage, colonized individuals exhibiting higher risk for endogenous infections, coupled with increased transmission rates through multiple means, such as contaminated hands, an especially important aspect when healthcare workers (HCWs) are concerned [2,3,4,5,6]. Several decolonization regimens are currently used worldwide in eradicating MRSA carriage in asymptomatic individuals; however, efficacy results exhibit increased variation in available sources [7,8,9]. Moreover, recent studies suggest MRSA decolonization treatment may have persistent altering effects on mucosal microbiota, suggesting that the disruption of normal nasal microbiota following decolonization treatment may require restoration of commensal microbial communities [10].

Despite thorough implementation of infection prevention and control protocols in healthcare settings, occasional transmission events may still occur, particularly in environments involving frequent patient contact and trainee rotations. MRSA carriage rates show increased local variation in different cohorts, either among patients or HCWs, highlighting the need for local surveillance in hospitals in order for tailored infection prevention and control measures to be instated, as, on the other hand, hospitalization is linked to an increased risk of MRSA carriage, thus contributing to this public health issue [11,12,13,14,15,16]. Results of formerly conducted studies in our setting show an 8.24% prevalence on MRSA carriage from all colonization sites in admitted patients, correlating with data from available studies in the literature, which exhibit high variation in accordance with clinic specifics, estimating a higher prevalence in at-risk patients such as end-stage renal disease or chronic ulcer patients, highlighting the complex dynamic of MRSA colonization and transmission [16,17,18,19,20,21].

Medical students remain underrepresented in conventional hospital-based statistics, yet they increasingly interact with patients in multiple healthcare settings, placing them as a key transmission link in both intra-hospital and inter-hospital transmission. Studies show a rather low prevalence of MRSA carriage among European medical students, under 1% in most available sources analyzing both clinical and preclinical study years, yet available data from other continents present higher asymptomatic colonization rates, ranging from 5.5% in Pakistan to 33.3% in an Ethiopian cohort [22,23,24,25,26,27,28].

Methodological factors may also influence MRSA recovery rates, as different collection methods show variable results in terms of bacterial growth. Pre-moistening of swabs has been hypothesized to improve bacterial recovery by increasing the adherence of microorganisms to the swab surface, thus improving recovery from relatively dry mucosal surfaces. However, nasal swab collection for MRSA carriage using the conventional dry, sterile cotton swab versus a swab pre-moistened in sterile saline did not show statistically significant differences [29].

This study aimed to provide an updated overview of the prevalence of MRSA carriers among clinical year medical students at the largest Romanian medical university (a cohort exposed to multiple healthcare settings throughout study years) and the correlations with risk factors. In addition, it was aimed to evaluate whether pre-moistening the nasal swab before sample collection influenced the frequency of MRSA isolation.

## 2. Materials and Methods

### 2.1. Study Design

This cross-sectional study was conducted in the Microbiology Laboratory of the ‘Prof. Dr. Matei Balș’ National Institute of Infectious Diseases in Bucharest, Romania, during February–March 2026 and focused on students enrolled in the clinical years (years of study 4–6) of the Faculty of Medicine within the ‘Carol Davila’ University of Medicine and Pharmacy in Bucharest, Romania. Participation was completely voluntary; students were distributed a registration questionnaire which contained questions regarding demographics and risk factors for MRSA carriage and inner-hospital hygiene habits (Appendix A). The sample size was calculated using validated formulas for statistical significance, estimating the prevalence of MRSA nasal carriage at 6%, targeting for a 95% confidence interval with 5% margin of error. Informed consent was obtained from all subjects involved in the study. The investigators provided each participant with information about the procedure and data use and distributed information leaflets prior to sampling. The study was approved by the Institutional Ethics Committee of the ‘Carol Davila’ University of Medicine and Pharmacy.

### 2.2. Specimen Sampling

Sampling was performed according to clinical guidelines using sterile nasal swabs. A single swab was used per participant. Each swab was inserted into both nostrils of the subject, parallel to the hard palate, and rotated for approximately 30 s. In addition, a randomized comparative analysis was incorporated to evaluate the effect of swab pre-moistening on MRSA recovery rates. Thus, participants were randomly divided into two subgroups, by a computer-generated randomization—those who underwent standard dry swab collection and pre-moistened swab collection using sterile saline, respectively. The two groups were considered comparable, given that all participants met identical inclusion criteria (Table 1).

### 2.3. Bacterial Culture

All nasal swabs were inoculated immediately after collection on chromogenic MRSA agar (Brilliance MRSA 2 Agar, ThermoScientific™, Oxoid Deutschland GmbH, Wesel, Germany) and incubated for 24–48 h at 35–37 °C in aerobic atmosphere. Plates were then visually read and interpreted. Isolates that showed characteristic growth on MRSA chromogenic medium (blue-pigmented colonies) were subjected to further testing and identification.

### 2.4. Bacterial Identification

All presumptive MRSA colonies were subjected to coagulase production testing using the Staphytect Plus kits (Oxoid Ltd., Basingstoke, UK). In addition, bacterial identification was confirmed using MALDI-TOF (Matrix-Assisted Laser Desorption Ionization Time-of-Flight) mass spectrometry on the VITEK^®^2 MS PRIME system (bioMérieux, Marcy-l’Étoile, France), software version 1.1, VITEK^®^MS IVD Database version 3.3.

### 2.5. Methicillin Resistance Confirmation

*Staphylococcus aureus* isolates were further subjected to methicillin resistance testing, using the disk diffusion method on Mueller Hinton agar (MHE, bioMérieux, France), with Cefoxitin 30 μg disks (FOX, Oxoid Ltd., Basingstoke, UK). Results were interpreted by reporting to the EUCAST (European Committee on Antimicrobial Susceptibility Testing) guidelines version 16.0, with isolates considered resistant when an inhibition zone of less than 22 mm in diameter was observed.

### 2.6. Quality Control

Quality control is constantly performed for all reagents, in accordance with laboratory’s internal protocol. For chromogenic MRSA media, quality control was performed using *Staphylococcus aureus* ATCC 29213 and *Staphylococcus aureus* ATCC 33591, respectively. For the coagulase testing kits, quality control was performed using the negative control reagent provided in the package, in addition to *Staphylococcus aureus* ATCC 25923 as positive control. *Staphylococcus aureus* ATCC 29213 was used for the quality assurance of Cefoxitin disks.

### 2.7. Data Storage and Analysis

All samples were processed anonymously after sample collection, as each swab received an individual sample code. All data were subsequently stored digitally and analyzed in a database assigned to the project, using Microsoft Excel version 16.66.1 (2022, Microsoft). The Chi-square test (two-tailed) was used for categorical variables, with results considered statistically significant at *p*-value < 0.05. In several analyses, Fisher’s exact test was used when Chi-square assumptions were unfulfilled, given the low expected frequencies. The Wilson method was used for confidence interval calculations.

## 3. Results

### 3.1. Cohort Demographics

A total of 156 clinical-year medical students were included in the study, pertaining to the 4th (21.15%, n = 33), 5th (12.17%, n = 19) and 6th (66.66%, n = 104) study years. Fifty-three participants were male (33.97%) and 101 female (64.74%), with two subjects opting out of mentioning their gender. The median age of the cohort was 24 years, with a standard deviation of ±1.63 years. Division into the two subgroups regarding sample collection technique led to similar characteristics among cohorts (Table 1).

### 3.2. MRSA Prevalence

Nine students (5.76%, 95% CI: 3.05–10.58%) were identified to be asymptomatic nasal carriers of MRSA, with no statistically significant differences between the two subgroups undergoing different sampling methods—5.00% (n = 4/80) among the conventional dry swab sampling subgroup, compared to 6.57% (n = 5/76) in the pre-moistened swab sampling subgroup, *p* = 0.74.

### 3.3. Hospital Exposure and Inner-Hospital Hygiene Habits

When questioned about hospital exposure, participants were required to answer questions regarding the number of clinical rotations attended by the moment of completing the questionnaire and the number of separate hospitals attended during the mentioned clinical rotations. Most respondents (67.30%, n = 105) reported attending more than 15 clinical rotations by the time of response, 9.61% (n = 15) reported 10–15 rotations attended, 17.30% (n = 27) 5–10 rotations and 5.76% (n = 9) respondents attended less than 5 rotations by the time of survey completion. Regarding the number of hospitals, the same percentage was observed with visitors of less than 5 hospitals (5.76%, n = 9), while 37.82% (n = 59) visited 5–10 hospitals, 25.00% (n = 39) reported 10–15 hospitals visited and 31.41% (n = 49) visited more than 15 hospitals during clinical rotations. Moreover, 96 students (61.53%) reported participating in extracurricular activities in hospital environments, such as volunteering or research activities.

Questions regarding inner-hospital hygiene habits focused on hand washing practices and additional equipment usage, depending on each hospital ward specifics (e.g., hand gloves, facial masks, single-use overcoats, etc.). The majority of respondents (67.30%, n = 105) reported always washing or disinfecting their hands before and after patient contact, with 31.14% (n = 49) mentioned this habit occurring on most times and two subjects reporting occasional hand hygiene in relation to patient contact. Students mentioned the usage of ward-specific additional equipment rarely (2.56%, n = 4), occasionally (9.61%, n = 15), in most cases (54.48%, n = 85) and always (33.33%, n = 52).

### 3.4. Correlations Between MRSA Carriage and Hospital Exposure

In relation to hospital exposure, relative risk (RR) for MRSA carriage was calculated for multiple parameters, with no statistically significant association identified. In relation to the number of clinical rotations attended, the relative risk of carrying MRSA in students who participated in more than 15 clinical rotations compared to those participating in fewer than 10 rotations was RR = 0.57 (95% CI: 0.14–2.27, *p* = 0.42). Students who visited more than 15 hospitals had a higher relative risk of being carriers of MRSA compared to their peers who visited fewer than 10 hospitals during clinical rotations (RR = 1.38; 95% CI: 0.29–6.58; *p* = 0.12). However, participation in extracurricular activities in hospital environments did not play a statistically significant role in MRSA carriage (RR = 0.57; 95% CI: 0.14–2.27; *p* = 0.73). Table 2 presents detailed statistics regarding parameters in relation to hospital exposure. Additional questionnaire variables, including recent antibiotic use and immunosuppressive conditions were not included in the inferential analyses due to the simplified binary structure of questionnaire items, which limited detailed assessment of exposure and relevance.

## 4. Discussion

This study provides an updated version of data on the presence of MRSA carriage among Romanian medical students in the clinical years at the largest medical university in the country and is the first paper to provide such data on Romanian students. Medical students are constantly involved in patient care and face exposure to multiple healthcare environments, thus they can serve as sources of transmission not only interpersonally, but also between hospitals, given that clinical rotations are organized in numerous hospitals in Bucharest, sometimes the students visiting several hospitals during a day.

The 5.76% MRSA prevalence in this study appears higher than other available sources investigating similar European cohorts—Cirkovic et al. identified a 0.37% MRSA prevalence in medical students from Belgrade, Serbia, Cavaco-Silva et al. reported a 0.86% prevalence in Portugal, while Gualdoni et al. detected no MRSA carriage among medical students in Vienna [22,23,24,25]. However, no recently published studies are available, suggesting this data may not be currently applicable. Compared to studies in Africa or South America, results of this study show lower carriage rates, with Reyes et al. reporting an 8.4% MRSA nasal carriage in second-year Colombian medical students, while the analysis published by Shume et al. revealed a 4.8% carriage rate in all university students, with a 33.3% prevalence among medical students from Haramaya, Ethiopia [26,27]. Another similar study, conducted by Taqweem et al. in Pakistan, illustrated a 5.5% MRSA nasal carriage prevalence among a cohort of 1200 students [28]. There is a lack of comparable data from other Romanian centers regarding medical students or medical staff, however the present study reported a higher prevalence when comparing to a meta-analysis investigating MRSA carriage in HCWs in Europe and North America, which described the pooled MRSA colonization rate to be 1.8% [11,30]. Therefore, the potential role played by medical students in the spread of MRSA should not be underestimated, even if carrier prevalence remains at low rates. However, the direct comparison between these studies should be cautiously interpreted due to differences in healthcare systems, methodologies and participant characteristics.

Even though increased hospital exposure could theoretically increase the risk of MRSA colonization, the presented results do not demonstrate significant associations with any of the evaluated parameters, calculations obtaining wide confidence intervals. This may be explained by the relatively small number of MRSA-positive subjects and limited statistical power, although the sample size calculations for prevalence estimation were adequate for prevalence estimation. Therefore, larger studies are needed to assess whether cumulative hospital exposure may be a risk factor for MRSA carriage. Moreover, although most participants reported regular use of ward-specific protective equipment, the present study design does not allow for the assessment of effectiveness of individual preventive measures against MRSA carriage.

From a methodological point of view, the lack of statistically significant differences in MRSA isolation between pre-moistened and dry swabs collection subgroups suggests that standard dry swab sampling remains a valid collection method for nasal pathogen carrier screening, although larger studies are needed, given the wide confidence intervals due to the limited sample size.

### Limitations

Several limitations of the study should be mentioned, although these are commonly encountered in cross-sectional studies. First, the single-center design limits the generalizability of the results, but the importance of surveillance of local asymptomatic carriers for better understanding of microorganism transmission and prevention and control of healthcare-associated infections remains crucial. Second, we analyzed only nasal carriage, thus overlooking important extra-nasal areas, such as the skin or pharynx. However, this was done to increase the compliance of the study participants. The lack of molecular confirmation is another limitation; however, MRSA characterization was performed according to EUCAST standards. Although the calculated sample size was sufficient to obtain statistically applicable results, enrolling a larger number of participants would have been potentially useful detecting subtle differences in sampling performance. In addition, all participant answers were self-reported, thus subject to response bias. Lastly, this study did not discern between patient-student transmission and inter-student transmission, considering the cross-sectional design, yet student-to-student transmission routes outside hospital settings may also contribute to MRSA carriage. Other potentially important variables including previous hospitalization or household healthcare exposure were not assessed. Despite the limitations, this study contributes to the scarce evidence regarding MRSA carriage among medical students in Romania.

## 5. Conclusions

Asymptomatic carriers of MRSA may represent an important source of intrahospital transmission from healthcare workers to patients and beyond. This study focused on an often-overlooked category of medical personnel—medical students, who may represent a relevant population in MRSA transmission dynamics. The prevalence of asymptomatic nasal carriage in this cohort is 5.76%, with no statistical significance regarding the risk factors assessed or the difference between collection methods.

## Figures and Tables

**Table 1:** Baseline characteristics of the two subgroups. Subgroup 1 underwent conventional dry swab sampling, while Subgroup 2 was subjected to pre-moistened swab collection.

Characteristic		Subgroup 1 (n = 80)	Subgroup 2 (n = 76)	*p*-Value
Age		24 years, ±1.68	24 years, ±1.43	-
Gender	Male	27 (33.75%)	26 (34.21%)	0.95
	Female	52 (65.00%)	49 (64.47%)	0.94
Study year	4th	20 (25.00%)	13 (17.10%)	0.22
	5th	12 (15.00%)	7 (9.21%)	0.26
	6th	48 (60.00%)	56 (73.68%)	0.07

**Table 2:** Correlations between hospital exposure and extracurricular activities partaking, intrahospital hygiene practices and MRSA carriage.

Number of Hospitals Visited	Have Taken Part in Extracurricular Activities N (%)	Always Practicing Hand Hygiene Before and After Patient Contact N (%)	Regular Use of Ward-Specific Additional Equipment N (%)	MRSA Carriage N (%)
<5 (n = 9)	7 (77.77%)	6 (66.66%)	3 (33.33%)	0 (0.00%)
6–10 (n = 59)	40 (67.79%)	38 (64.40%)	17 (28.81%)	3 (5.08%)
11–15 (n = 39)	26 (66.66%)	27 (69.23%)	10 (25.64%)	1 (2.56%)
>15 (n = 49)	23 (46.93%)	34 (69.38%)	22 (44.89%)	5 (10.20%)

## Data Availability

The complete datasets are available upon reasonable request from the corresponding author.

## Abbreviations

The following abbreviations are used in this manuscript:

MRSA	Methicillin-resistant *Staphylococcus aureus*
HCWs	Healthcare workers
MSSA	Methicillin-susceptible *Staphylococcus aureus*
MALDI -TOF	Matrix-Assisted Laser Desorption Ionization Time-of-Flight
EUCAST	European Committee on Antimicrobial Susceptibility Testing

## Appendix A

**Table A1:** Complete questionnaire structure.

Question Number	Question	Possible Answers (Where Applicable)
1	Name and surname	
2	Age	
3	Gender	
4	Email address and phone number (for sample collection scheduling)	
5	How many clinical rotations have you attended by the time of completing this questionnaire (including Semiology)	A. <5 B. 6–10 C. 11–15 D. >15
6	How many hospitals have you visited in total during said clinical rotations?	a. <5 b. 6–10 c. 11–15 d. >15
7	Have you taken antibiotics during the last 3 months	a. Yes b. No
8	Do you have any history of chronic immune-suppressing conditions (e.g., HIV infection, autoimmune diseases in immunosuppressing treatment, etc.)	a. Yes b. No
9	Have you taken part in extracurricular activities in medical institutions (e.g., hospitals, private clinics, ambulance services)? (e.g., volunteering, research activities)	a. Yes b. No
10	How frequently do you wash/disinfect your hands during hospital rotations?	A. Always before and after having contact with the patient B. Most of the time C. Occasionally D. Rarely
11	How frequently do you use additional protective equipment (e.g., gloves, single-use overcoats, facial masks) according to the ward specifics?	A. Always B. Most of the time C. Occasionally D. Rarely
